# The perceived social support of parents having bipolar disorder impacts their children’s mental health: a 10-year longitudinal study

**DOI:** 10.1186/s40345-024-00349-4

**Published:** 2024-07-27

**Authors:** Florencia Trespalacios, Ariel Boyle, Lisa Serravalle, Sheilagh Hodgins, Mark A. Ellenbogen

**Affiliations:** 1 Centre for Research in Human Development, Department of Psychology, Concordia University, Montréal, QC H4B 1R6 Canada; 2 Département de Psychiatrie, Université de Montréal, Montréal, Canada; 3 Department of Clinical Neuroscience, Karolinska Institute, Stockholm, Sweden

**Keywords:** High-risk children, Developmental psychopathology, Protective factors, Bipolar disorder, Social support

## Abstract

**Background:**

The offspring of parents with bipolar disorder (OBD) are at higher risk of developing psychopathology than the offspring of parents with no affective disorder (control). In addition to genetic predisposition, childhood adversity and a stressful family environment are important risk factors for the OBD. Protective factors in parents, such as social support and coping strategies, may buffer the effects of stress on at-risk children. This study tested whether parents’ social support and coping style attenuated the link between risk status (OBD vs. control) and psychopathology in offspring.

**Methods:**

During offspring’s middle childhood, parents underwent a diagnostic interview and completed social support and coping style questionnaires. Sixty-nine OBD (39 female) and 69 control (29 female) offspring between ages 13 and 29 completed a diagnostic interview approximately 10 years later.

**Results:**

Parents’ social support satisfaction moderated the link between offspring risk status and their development of substance use disorder (SUD) symptoms (F(1,131) = 5.90, *p* = .017). Parents’ social network size moderated the link between offspring risk status and their development of anxiety and depression symptoms in an unexpected direction (F(1,131) = 5.07, *p* = .026). No effects of parents’ coping style were found.

**Conclusions:**

Among the OBD, having parents with greater social support satisfaction and, unexpectedly, a smaller social network buffered their development of SUD and depression and anxiety symptoms by early adulthood. Parents’ social support may thus have a protective function for children in these high-risk families.

**Supplementary Information:**

The online version contains supplementary material available at 10.1186/s40345-024-00349-4.

## Background

Bipolar Disorder (BD) is a chronic mental disorder that impairs cognitive and psychosocial functioning, and negatively affects quality of life (Grande et al. 2016). BD poses an important societal burden, including the high costs of disability, treatment, and comorbid mental and physical conditions (Conus et al. 2014). Moreover, BD in a parent is associated with impaired family functioning that in turn is associated with difficulties among their children (Ellenbogen and Hodgins 2004; Serravalle et al. 2020). Multiple studies report that offspring of parents with BD (OBD), relative to offspring of parents with no affective disorder (control), are at elevated risk for internalizing and externalizing problems in childhood, and developing an affective (i.e., major depressive disorder and BD), anxiety, and substance use disorder in adulthood (Birmaher et al. 2009, 2021; Iacono et al. 2018; Nijjar et al. 2014; Rasic et al. 2014; Sandstrom et al. 2020). One major vulnerability factor for the OBD is the high heritability of BD, which is estimated to account for 85% of the variance in twin studies (McGuffin et al. 2003). However, the negative outcomes observed in the OBD are still best conveyed through complex interactions between genetic and environmental factors (Brietzke et al. 2012).

Previous studies have identified environmental risk factors in families having a parent with BD, including suboptimal parenting practices, poor communication strategies among parents, family conflict, low family cohesion, and a lack of organization and consistency in the home (Calam et al. 2012; Chang et al. 2001; Ellenbogen et al., 2004; Iacono et al. 2018; Stapp et al. 2020; Vance et al. 2008). In turn, the stressful home environment in these families is associated with an elevated risk of emotional, behavioral, and interpersonal difficulties among the OBD (Bella et al. 2011; Iacono et al. 2018; Ostiguy et al. 2012; Whitney et al. 2013). Indeed, anxiety and sleep problems in childhood appear to be an early marker of risk among the OBD, that precede the emergence of subthreshold affective symptoms and substance use problems in adolescence, followed by the onset of an affective disorder (Duffy et al. 2014, 2019). Research has focused mainly on risk factors and developmental trajectories in the OBD, rarely identifying protective factors that have the potential to improve current functioning and longer-term outcomes.

Social support is a well-established contributor to greater overall well-being, buffering against psychological distress, depression and anxiety, and even reducing the risk of mortality (Cohen 2004; Gariépy et al. 2016; Espinosa and Rudenstine 2020; Holt-Lunstad et al. 2010). Relative to parents without a mental disorder, parents with BD and their intimate partners report smaller social networks, less social contact, and lower satisfaction with their own social support (Serravalle et al. 2020). Low perceived social support in individuals with BD has been linked with lower medication compliance, increased stress, and more depressive episodes over the course of a year (Boyers & Rowe, 2018; Cohen et al. 2004), potentially exposing their offspring to a more stressful and unpredictable family environment. Longitudinal investigations have shown that poor social support among parents is a risk factor that promotes the development of psychopathology in their offspring (Ashman et al. 2008; Barker et al. 2012). By contrast, studies that find higher levels of social support reported by parents are linked to more optimal parenting practices and better psychological adjustment in their offspring (Hughes et al. 2020; Nunes et al. 2021; Waylen and Stewart-Brown 2010). Taken together, it is thus plausible that having parents with high perceived social support is a protective factor that may buffer the OBD’s high risk for mental disorders and other negative outcomes.

In addition to social support, a person’s ability to adaptively cope with stressors is another factor that promotes physical and psychological well-being (Marroquín et al. 2017; Skinner and Zimmer-Gembeck 2015, 2016). Endler and Parker (1994) identified three dimensions of coping in response to stressors: task-oriented (i.e. attempt to problem-solve), emotion-focused (i.e. attempt to self-regulate emotional response), and avoidant-oriented (i.e. attempt to distract oneself). Emotion-focused and avoidant-oriented coping are generally associated with greater symptoms of psychopathology, whereas task-oriented coping is linked to lower psychological distress (Endler et al., 1994; Higgins and Endler 1995; Skinner et al., 2016). Individuals with BD, as well as their intimate partners, are more reliant on maladaptive coping strategies such as emotion-oriented coping compared to persons with no mental disorder (Fletcher et al. 2013; Moon et al. 2014; Serravalle et al. 2020). Among parents with BD, those who rely more on emotion-focused coping foster stressful family environments that can negatively influence their offspring’s psychosocial functioning, relative to those who engage in more task-oriented coping (Borowiecka-Karpiuk et al. 2014; Ellenbogen et al., 2004). Moreover, the effect that parents’ coping strategies may have on OBD could be further exacerbated by their likelihood of adopting or modelling their parents’ strategies (Jones et al. 2006; Liga et al. 2020; Nijjar et al. 2014). Thus, the coping strategy that parents with BD rely on could be either an important risk or protective factor for the development of psychopathology in the OBD.

To date, there are no longitudinal studies assessing whether parents’ social support and effective coping serve as a protective factor for OBD. Thus, the present study aimed to determine whether higher levels of social support and the use of effective coping strategies by parents when their offspring were in middle childhood were associated with lower levels of mental health problems among their offspring ten years later. We hypothesized that parents’ social support when their children were in middle childhood would moderate the relationship between risk status (OBD vs. control) and the offspring’s development of psychopathology symptoms. That is, parents’ higher levels of social support (i.e., number of contacts and satisfaction with support received) was expected to attenuate the development of symptoms of depression, anxiety, and substance use disorder (SUD) among the OBD, but not control offspring. We also hypothesized that parents’ coping style would moderate the relationship between risk status and the development of psychopathology symptoms in their offspring. We predicted that having parents who used more task-oriented and less emotion-oriented coping would attenuate the development of symptoms of depression, anxiety and SUD among the OBD, but not control offspring.

## Methods

### Participants

A total of 105 families were recruited into a longitudinal study for which data collection occurred at two time points – the first between 1996 and 1998 (time 1), and the second approximately 10 years later (time 2). Families fluent in English or French had at least one biological child between 4 and 14 years of age who had been raised and educated in Canada. Families were excluded if a parent or child had a chronic physical condition or handicap, or an IQ below 70. Families in which at least one parent had a diagnosis of BD were recruited from psychiatric outpatient clinics in Québec, as well as from advocacy and support groups. Control group families, in which neither parent had an affective disorder, were recruited from physicians’ offices and community organizations within the same neighbourhoods as those with a parent having BD. At time 1, parents’ mental health status was assessed.

Of the 105 families assessed at time 1, 80 (45 families having a parent with BD, 35 control group families) completed the assessment at time 2, indicating an attrition of 24%. Offspring who did not participate in the time 2 follow-up assessment did not differ from those who did on time 1 ratings of childhood behavior problems and IQ. The sample for this study therefore included 138 offspring (69 OBD and 69 control) between the ages of 13 and 29, from these 80 families. Sixty-eight of the offspring (29 control, 39 OBD) were female.

### Measures

#### Parent assessment at time 1

##### Parents’ diagnoses

The Structured Clinical Interview for DSM-III-R (SCID-I; Spitzer et al. 1992) is a semi-structured diagnostic interview used to assess mental disorders in adults. Independent inter-rater agreements were computed for 15% of the interviews. Agreement between clinicians was excellent, as indicated by the kappa coefficients for diagnoses of bipolar disorder, 1.0, and other mood disorders 1.0 (lifetime and current).

##### Social support

The Arizona Social Support Interview Schedule (ASSIS; Barrera 1980) is a 30-item semi-structured interview assessing the size of participants’ social network and their satisfaction with their social support. Social support could be provided by any person identified by the participant, including family members, friends, co-workers, a family doctor, etc. The study aimed to assess family-wide social support as a protective factor for children, thus we used the mean ASSIS score of all parents in each family. Internal consistency (Cronbach’s α = 0.74 − 0.78) for the ASSIS was adequate (Barrera et al., 1980).

##### Coping

The adult version of the Coping Inventory for Stressful Situations (CISS; Endler and Parker 1994) is a 48-item self-report questionnaire. It assesses the extent to which individuals engaged in different coping activities following stressful situations, using a five-point scale ranging from 1 (*Not at all*) to 5 (*Very much*). Standardized *T* scores for three primary styles of coping (task-oriented, emotion-focused, and avoidance-oriented) were obtained. For this study, we used the mean task- and emotion-oriented coping scores of all parents in each family. High internal consistency (Cronbach’s α = 0.78 − 0.88) and temporal stability have been reported for the CISS (Brands et al. 2014). Data for coping styles was missing for parents in one OBD family.

#### Offspring assessment at time 1

##### Offspring time 1 mental health

The Child Assessment Schedule (CAS; Hodges et al. 1982a) and Parent Interview CAS (Graham and Rutter 1968) are semi-structured diagnostic interviews conducted with the child (not reported here) and parent(s), respectively. It was administered by a trained clinical psychologist and assessed DSM-III (American Psychiatric Association 1980) diagnoses. In this study, a total score representing the number of current symptoms across all affective, anxious, and disruptive behavior disorders was created. There is substantial evidence of interrater reliability and internal consistency (Hodges et al. 1982b), and diagnostic agreement between child and parent informants has also been established (Verhulst et al. 1987). Given that 21% of the CAS parent-report data was missing, regression imputation was used to replace missing values.

#### Offspring assessment at time 2

##### Offspring time 2 mental health

The Kiddie Schedule for Affective Disorders and Schizophrenia – Present and Lifetime version (K-SADS; Kaufman and Schweder 2004) was used to assess mental disorders in offspring under 18 years of age, and the SCID-I for DSM-IV-TR (First et al. 2002) was used for those 18 years and above. The number of current (i.e., within the previous month) and lifetime symptoms of depression, anxiety and SUD were assessed. Both diagnostic instruments demonstrate good psychometric properties (Basco et al. 2000; First et al. 2002; Kaufman et al., 2004). Interrater reliability obtained for 15% of interviews was excellent (k = 0.82).

### Procedure

Following a telephone screening, all parents completed the SCID-I interview, the CAS and the ASSIS, which were administered by a trained interviewer in the laboratory or at their homes. They also completed a battery of questionnaires, including the CISS (see Serravalle et al. 2020 for full data collection details). Parents with BD were required to be euthymic at the time 1 assessment. If not, the assessment was delayed until clinical remission of the current episode was achieved. Approximately 10 years later, informed consent was obtained directly from adult offspring, and from parents for the adolescent offspring. Offspring were then scheduled to come into the laboratory to undergo a diagnostic assessment (K-SADS or SCID-I), conducted by a trained interviewer. Offspring were compensated $150 CAD at time 2 for participating in the full data collection.

Descriptive data of parents, their partners and their offspring are reported in Ellenbogen and Hodgins (2004), Nijjar and colleagues (2014) and Serravalle and colleagues (2020). In addition, previous studies on this cohort of offspring have reported on daytime cortisol and cortisol reactivity (Ellenbogen et al. 2006, 2010, 2013; Ellenbogen and Hodgins 2009; Ostiguy et al. 2011), interpersonal functioning (Ellenbogen et al. 2013; Linnen et al. 2009; Ostiguy, 2012), sexual risk behaviors (Nijjar et al. 2014, 2016), chronic stress (Ostiguy et al. 2009), family functioning (Ellenbogen and Hodgins 2004), and parenting practices (Iacono et al. 2018).

### Statistical analyses

Data were screened and corrected for outliers and distributional anomalies that violated statistical assumptions. Due to the low number of diagnoses in the offspring, clinical and sub-clinical symptom counts of depression and anxiety symptoms combined (internalizing problems) and SUD symptoms (externalizing problems) were used for these analyses. Ordinary least squares (OLS) regressions were computed to assess whether parents’ social network size, social support satisfaction, and use of task- and emotion-oriented coping during the offspring’s middle childhood moderated the relationship between risk status (OBD vs. control) and offspring symptoms of anxiety and depression, and SUD in late adolescence and early adulthood (see Supplementary Fig. 1). The regression models estimated offspring symptoms of anxiety and depression and SUD at time 2, with offspring risk status, the time 1 moderator, and the interaction between these variables as predictors. To control for between-group differences across families with a parent having BD and control families, parents’ average education level, as a proxy of socioeconomic status, clinical symptoms in offspring at time 1 using the CAS score (parent-report), and offspring age at time 2 (given the significant difference between groups) were included as covariates in all models. Analyses were conducted using SPSS (version 27) and the PROCESS macro (version 4.0; Hayes & Little, 2018) for SPSS. Significant interactions (risk status X moderator) were followed up with the Johnson-Neyman technique to assess the regions of significance of the conditional effects along the distribution of values of the continuous moderators. PROCESS conducts tests of significance by constructing 95% bias-corrected confidence intervals. If the confidence intervals do not include zero, the interaction is statistically significant at the 0.05 level. The bootstrap sample was set at 5000 iterations.

There was one notable outlier in the social network size variable, scoring more than 3 standard deviations above the overall mean of scores. Given that it strongly deviated from the mean, all analyses were conducted after excluding the outlier, and the pattern of results was maintained. We therefore decided to keep this participant in the analyses, since the value of their social network size is nonetheless possible and valid.

Given that there are robust sex differences in the development of mental disorders in youth (Kistner 2009), we examined whether offspring sex moderated the above analyses. Offspring sex did not moderate any of the analyses and thus was excluded from the analyses.

To determine whether violations of independence (children nested within families) might have influenced the findings, all regression analyses were repeated using multilevel modeling with the program Hierarchical Linear Modeling (version 8.0; Raudenbush et al. 2019). Multilevel modeling can accommodate for violations of the statistical assumption of independence in sampling.

## Results

### Descriptive data

At time 1, there was no difference in mean ages of control offspring (M = 7.77, SD = 2.35) and OBD (M = 8.45, SD = 2.44, t = -1.46, *p* = .174). At time 2, control offspring (M = 18.80, SD = 3.34) were slightly younger than the OBD (M = 20.2, SD = 3.45, t = -2.46, *p* = .015). The parents with no affective disorder had attained higher levels of education (M = 15.84, SD = 2.28) than the parents with BD and their partners (M = 14.43, SD = 2.58; t = 3.41, *p* = .001). The number of psychiatric symptoms in the offspring at time 1, as reported by their parent, were higher for the OBD (M = 8.00, SD = 7.52) than control offspring (M = 4.84, SD = 6.07; t = -2.72, *p* = .007). Group differences for parents’ social support and coping variables are shown in Table 1.

**Table 1:** Comparisons between control offspring and OBD for time 1 parent reports of social support and coping strategies, and time 2 offspring symptoms and diagnoses of mental disorders

	Control	OBD	
	Time 1 (1996–1998)		
*n*	69	69	
	***M*** **(SD)**	***M*** **(SD)**	***t***
Parents’ social network size^*a*^	17.55 (10.42)	11.81 (7.10)	3.78**
Parents’ social support satisfaction^*a*^	27.96 (2.34)	25.66 (2.99)	5.03**
Parents’ task-oriented coping^*b*^	52.71 (5.66)	48.34 (6.81)^*c*^	4.08**
Parents’ emotion-oriented coping^*b*^	47.33 (5.77)	51.65 (7.25)^*c*^	-3.86**
	Time 2 (2006–2008)		
*n*	69	69	
**Offspring variables**	***M*** **(SD)**	***M*** **(SD)**	***t***
Depression & anxiety symptoms^*d*^	4.39 (5.12)	6.26 (6.50)	-1.88
SUD symptoms^*d*^	1.62 (4.41)	4.30 (6.76)	-2.76**
	***n*** **(%)**	***n*** **(%)**	
At least one ***current*** diagnosis^*d*^	15 (21.7)	28 (40.6)	-2.42*
Affective disorder^*d*^	0 (0)	4 (5.8)	-2.05*
Anxiety disorder^*d*^	10 (14.5)	16 (23.2)	-1.31
SUD^*d*^	4 (5.8)	13 (18.8)	-2.36*
At least one ***lifetime*** diagnosis^*d*^	27 (39.1)	46 (66.7)	-3.35**
Affective disorder^*d*^	8 (11.6)	22 (31.9)	-2.96**
Anxiety disorder^*d*^	13 (18.8)	21 (30.4)	-1.54
SUD^*d*^	8 (11.6)	24 (34.8)	-3.33**

control = offspring of parents with no affective disorder

OBD = offspring of parents with bipolar disorder

*a* From the Arizona Social Support Interview Schedule

*b* From the Coping Inventory of Stressful Situations

*c n* = 68; data missing for one family

*d* From the Structured Clinical Interview for DSM-IV-TR or Kiddie Schedule for Affective Disorders and Schizophrenia

*p < .05. **p < .01

At time 1, of the 66 parents in the control families, approximately 3% received a current or lifetime anxiety disorder diagnosis, 1.5% received a current or lifetime alcohol abuse or dependence diagnosis and 4.6% a drug abuse or dependence diagnosis. Of the 92 parents in families with a parent having bipolar disorder, approximately 17.4% received a current or lifetime diagnosis of major depressive disorder, 10.9% received a current or lifetime anxiety disorder diagnosis, 15.2% received a current or lifetime alcohol abuse or dependence diagnosis and 9.8% a drug abuse or dependence diagnosis.

At time 2, 43 offspring (15 control, 28 OBD) met DSM-IV-TR criteria for at least one *current* diagnosis, and 73 offspring (27 control, 46 OBD) met criteria for at least one *lifetime* diagnosis (see Table 1 for rates per diagnostic category). For more information on offspring diagnoses at time 2 (i.e., type of anxiety disorder), see Nijjar and colleagues (2014). There was a statistically significant difference between OBD and control offspring’s mean level of SUD symptoms, but not symptoms of an affective or anxiety disorder. Pearson correlations between all variables are shown in Table 2.

**Table 2:** Pearson correlation coefficients for study variables

Variable	1	2	3	4	5	6	7	8	9	10
Time 1 assessment										
1. Offspring risk status^*a*^	−	− 0.28**	0.23**	− 0.31**	− 0.40**	− 0.33**	0.32**	0.21*	0.16	0.23**
2. Parents’ mean education level		−	− 0.14	0.30**	0.18*	0.26**	− 0.32**	− 0.06	− 0.03	− 0.22*
3. Offspring psychiatric symptoms at time 1			−	− 0.06	0.01	− 0.11	0.11	− 0.07	0.30**	0.08
4. Parents’ social network size				−	0.27**	0.27**	− 0.22**	− 0.09	− 0.04	− 0.22**
5. Parents’ social support satisfaction					−	0.29**	-25**	− 0.01	− 0.02	− 0.19*
6. Parents’ task-oriented coping						−	− 0.30**	− 0.14	− 0.03	− 0.07
7. Parents’ emotion-oriented coping							−	0.07	0.07	0.12
Offspring outcomes at time 2										
8. Offspring age								−	0.09	0.18*
9. Depression & anxiety symptoms									−	0.21*
10. SUD symptoms										−

Note. *p < .05. **p < .01. SUD: substance use disorder

*a* Control = -1, OBD = 1.

### The effect of parents’ social support satisfaction at time 1 on the relation between offspring risk status and psychopathology at time 2

The results for all predictors in the OLS regression model predicting offspring depression and anxiety symptoms at time 2 are shown in Supplementary Table S1. The offspring risk status by parents’ social support satisfaction at time 1 interaction term was not a significant predictor (*b* = -0.15, *t*(131) = -0.80, *p* = .428) and inclusion of the interaction term did not lead to a significant increase in model fit (*R*^*2*^_*change*_ = 0.00, F(1, 131) = 0.63, *p* = .428).

**Fig. 1:**
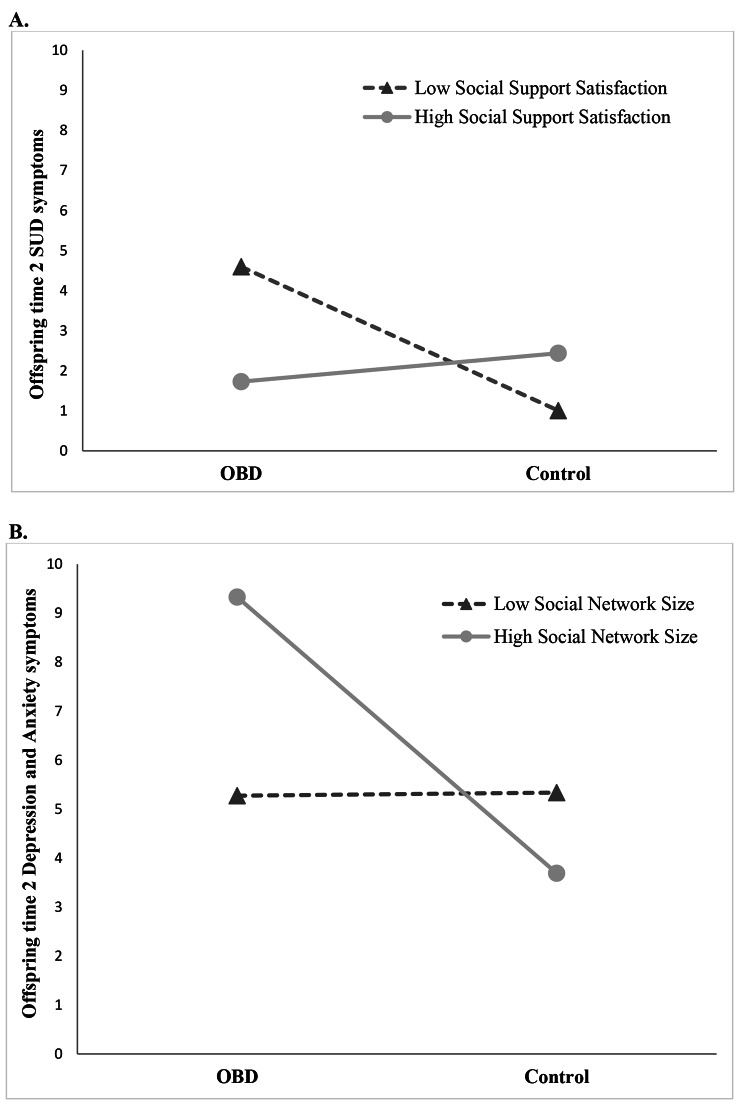
The figure depicts parents‘ satisfaction with their social network (A) and reported network size (B) as moderators of the relationship between risk status (having a parent with bipolar disorder or not) and time 2 outcomes (number of symptoms) in the offspring. Low and high social support are depicted as 1 standard deviation below and above the mean, respectively. **A**: Offspring of parents with bipolar disorder (OBD) with parents reporting high social support satisfaction developed fewer symptoms of subtance use disoders (SUD) at time 2 compared to OBD with parents reporting low satisfaction with their social support and control offspring. **B**: OBD with parents reporting a large social network developed more symptoms of major depressive disorder and anxiety disorders at time 2 compared to OBD with parents reporting a small network and control offspring

The results for all predictors in the OLS regression model predicting offspring SUD symptoms at time 2 are shown in Table 3. The offspring risk status by parents’ social support satisfaction at time 1 interaction term was a significant negative predictor of offspring SUD symptoms at time 2 (*b* = -0.45, *t*(131) = -2.43, *p* = .017). Inclusion of the interaction term led to a significant increase in model fit, *R*^*2*^_*change*_ = 0.038, F(1, 131) = 5.90, *p* = .017. Analyses of conditional effects of risk status at the 16th, 50th, and 84th percentile of parents’ social support satisfaction scores were conducted. As shown in Fig. 1A, robust group differences in SUD symptoms were found between OBD and control offspring when parents’ social support satisfaction was low (i.e., 16th percentile), *b* = 1.73, 95% CI [0.28, 3.18], *t*(131) = 2.36, *p* = .020, but this difference disappeared when parents’ social support satisfaction was average (i.e., 50th percentile), *b* = 0.39, 95% CI [-0.72, 1.50], *t*(131) = 0.69, *p* = .493, and high (i.e., 84th percentile), *b* = 0.51, 95% CI [-1.92, 0.90], *t*(131) = -0.72, *p* = .474. That is, the OBD with parents reporting lower social support satisfaction at time 1 had significantly more SUD symptoms at time 2 than control offspring whose parents reported similarly low levels of satisfaction with their social support. No group differences in offspring’s number of SUD symptoms at time 2 were observed when parents reported average or high social support satisfaction at time 1.

**Table 3:** Results of ordinary least squares (OLS) regression model predicting offspring substance use disorder symptoms at time 2 (Y) from offspring risk status (X), parents’ social support satisfaction at time 1 (W) and the X by W interaction term

Model	Estimate (b)	SE		95% CI		*p*
				LL	UL	
Risk status (**X**)	0.696	0.558		− 0.409	1.80	0.215
Parents’ social support satisfaction (**W**)	− 0.159	0.183		− 0.521	0.204	0.389
Offspring age	0.241	0.141		− 0.038	0.520	0.090
Mean parent education	− 0.284	0.197		− 0.674	0.107	0.153
Offspring time 1 psychiatric symptoms^*a*^	0.058	0.071		− 0.083	0.199	0.417
**X** by **W** interaction	− 0.430	0.183		− 0.792	− 0.069	0.020*

Note. * *p* < .05

*LL* = lower limit; *UL* = upper limit

*a* Number of clinical psychiatric symptoms reported by parents on the Child Assessment Schedule

### The effect of parents’ social network size at time 1 on the relation between offspring risk status and psychopathology at time 2

The results for all predictors in the OLS regression model predicting offspring depression and anxiety symptoms at time 2 are shown in Table 4. The offspring risk status by parents’ social network size at time 1 interaction was a significant positive predictor of depression and anxiety symptoms in the offspring at time 2 (*b* = 0.13, *t*(131) = 2.25, *p* = .026). Inclusion of the interaction term led to a significant increase in model fit, *R*^*2*^_*change*_ = 0.03, F(1, 131) = 5.07, *p* = .026. As shown in Fig. 1B, analyses of conditional effects revealed robust group differences in offspring depression and anxiety symptoms when parents’ social network size was large (i.e., 84th percentile), *b* = 2.20, 95% CI [0.365, 4.04], *t*(131) = 2.37, *p* = .019, but this difference disappeared when parents’ social network size was average (i.e., 50th percentile), *b* = 0.23, 95% CI [-0.852, 1.31], *t*(131) = 0.42, *p* = .674, and small (i.e., 16th percentile), *b* = -0.29, 95% CI [-1.56, 0.969], *t*(131) = -0.46, *p* = .646. That is, the OBD whose parents reported having a larger social network at time 1 had significantly more depression and anxiety symptoms at time 2 than control offspring whose parents had a similarly large social network. No group differences in offspring’s number of depression and anxiety symptoms at time 2 were observed when parents reported average or low social network sizes.

**Table 4:** Results of ordinary least squares (OLS) regression model predicting offspring depression and anxiety symptoms at time 2 (Y) from offspring risk status (X), parents’ social network size at time 1 (W) and the X by W interaction term

Model	Estimate (b)	SE		95% CI		*p*
				LL	UL	
Risk status (**X**)	0.652	0.539		− 0.415	1.72	0.229
Parents’ social network size (**W**)	0.051	0.06		− 0.068	0.17	0.398
Offspring age	0.122	0.144		− 0.162	0.406	0.398
Mean parent education	0.026	0.205		− 0.38	0.432	0.901
Offspring time 1 psychiatric symptoms^*a*^	0.229	0.071		0.087	0.37	0.002*
**X** by **W** interaction	0.133	0.059		0.016	0.249	0.026*

Note. * *p* < .05

*LL* = lower limit; *UL* = upper limit

*a* Number of clinical psychiatric symptoms reported by parents on the Child Assessment Schedule

The results for all predictors in the OLS regression model predicting time 2 offspring SUD symptoms are shown in Supplementary Table S2. The offspring risk status by parents’ social network size at time 1 interaction term was not a significant predictor of time 2 offspring SUD symptoms (*b* = -0.09, *t*(131) = -1.52, *p* = .132) and inclusion of the interaction term did not lead to a significant increase in model fit (*R*^*2*^_*change*_ = 0.01, F(1, 131) = 2.29, *p* = .132).

### The effect of parents’ use of task- and emotion-oriented coping at time 1 on the relationship between offspring risk status and psychopathology at time 2

The overall OLS regression models with task-oriented coping as the moderator did not show evidence of moderation. In the model predicting offspring depression and anxiety symptoms at time 2, the offspring risk status by parents’ task-oriented coping at time 1 interaction term was not a significant predictor of offspring depression and anxiety symptoms at time 2 (*b* = -0.05, *t*(130) = -0.62, *p* = .539). In the model predicting offspring SUD symptoms at time 2, the offspring risk status by parents’ task-oriented coping at time 1 interaction term was not a significant predictor of offspring SUD symptoms at time 2 either (*b* = -0.05, *t*(130) = -0.64, *p* = .522).

The overall OLS regression models with emotion-oriented coping as the moderator did not show evidence of moderation. In the model predicting offspring depression and anxiety symptoms at time 2, the offspring risk status by parents’ emotion-oriented coping at time 1 interaction term was not as significant predictor of offspring depression and anxiety symptoms at time 2 (*b* = -0.07, *t*(130) = -0.81, *p* = .422). In the model predicting offspring SUD symptoms at time 2, the offspring risk status by parents’ emotion-oriented coping at time 1 interaction term was not a significant predictor of offspring SUD symptoms at time 2 either (*b* = 0.01, *t*(130) = 0.08, *p* = .936).

### Additional analyses

To address the issue of non-independence, due to some offspring being nested within the same family, analyses for all eight models were repeated using multilevel modeling. In these analyses, we modelled intercept effects (number of symptoms at time 2) at level 1, considering the full sample of offspring (*n* = 138), and all predictors used in the previous analyses were entered at level 2, considering scores at the family level (*n* = 80). In accordance with the abovementioned results, risk group and the risk group by parents’ social support satisfaction interaction significantly predicted SUD symptoms in the offspring (*b* = -0.53, *t*(73) = -2.08, *p* = .041). Furthermore, risk group and the risk group by parents’ social network size interaction significantly predicted depression and anxiety symptoms in the offspring (*b* = 0.17, *t*(73) = 3.43, *p* < .001). The results of the other six models were not statistically significant, maintaining the same pattern as reported above.

To assess whether the results of each model were independent from the variance explained by the other moderator variables, we repeated the above analyses with the 3 additional moderators of the other models as covariates. The pattern of results remained the same for all the models, supporting the validity and strength of the abovementioned findings. Results of these analyses for the two statistically significant findings are shown in Supplementary Table S3 and Supplementary Table S4.

In addition, to account for the potential role of parent history of SUD at time 1 (diagnosis of lifetime or present alcohol and/or drug abuse or dependence) and severity of illness (using the social and occupational functioning assessment scale; SOFAS) on the outcomes, the analyses of each moderation model were also repeated adding these two variables as covariates. The pattern of results also remained the same.

Lastly, we sought to explore the potential moderating role of mean SOFAS ratings across parents in four of the moderation models. We conducted four moderated moderation models using model 3 in the PROCESS macro in SPSS to assess whether there exists a three-way interaction between offspring risk status (OBD vs. control), each of the social support variables in the parents (i.e., social support satisfaction and social network size) and SOFAS scores across parents, in predicting time 2 outcomes in the offspring (i.e., depression and anxiety, and SUD symptoms). We found no evidence of moderated moderation, indicating that the observed relationships between risk status, social support, and offspring outcomes were not significantly influenced by parents’ illness severity and functioning. Results of these analyses are shown in Supplementary Tables S5 to S8.

## Discussion

Two key findings emerged from the present study. First, as predicted, the OBD whose parents reported lower social support satisfaction while they were in middle childhood had significantly more SUD symptoms in late adolescence and early adulthood, relative to control offspring. When parents reported higher social support satisfaction, OBD and control offspring did not differ in their development of SUD symptoms. Growing up with parents who were more satisfied with their social support may therefore have acted as a protective factor for OBD. It might be surprising that the effect was specific to SUD symptoms, but it is well known that non-affective mental disorders, particularly substance use disorders, are common among OBD in adulthood (Carlson and Weintraub 1993). Second, and contrary to our hypothesis, having parents with a larger social network during middle childhood was associated with significantly higher depression and anxiety symptoms in late adolescence and young adulthood for the OBD, relative to the control offspring. No such group differences were found between offspring whose parents reported a smaller social network. Surprisingly, this suggests that having parents with a larger social network acted as a risk factor specifically for the OBD. Alternatively, having parents with a smaller social network acted as a protective factor for the OBD, who are at high risk for developing affective disorders.

The findings with respect to social support satisfaction are consistent with a study by Ashman and colleagues’ (2008) who showed that low social support in depressed mothers increased the likelihood of their children developing externalizing and internalizing problems relative to offspring of parents with no mental disorder. Perceived social support satisfaction predicts positive mental and physical health outcomes, and this relationship appears to be more common than findings linking positive outcomes to the size of one’s social network (VanderVoort 1999). Therefore, perhaps parental social support satisfaction helps buffer the OBD’s risk of developing externalizing problems, such as SUD, through its protective effects on the parents’ mental health (Cohen et al. 2004; Gariépy et al. 2016). Improvement in parents’ mental health may influence the development of psychopathology in offspring by improving parents’ interpersonal functioning, the quality of child supervision and the structure in the home; factors that play a key role in the development of externalizing problems in high-risk youth, including the OBD (Costello et al. 2003; Iacono et al. 2018; Serravalle et al. 2020). Overall, this is consistent with previous research showing that parental factors (i.e. personality characteristics, rearing practices and psychological functioning) have an important impact on the OBD’s psychological development (Ellenbogen et al., 2004; Iacono et al. 2018; Nunes et al. 2021). However, the specific mechanism(s) by which having parents with higher social support satisfaction attenuates the development of SUD symptoms in the OBD are still unknown (Klimes-Dougan et al. 2010).

The unexpected finding that the OBD, relative to control offspring, whose parents reported the largest social networks developed more depression and anxiety symptoms, and that the OBD and control offspring whose parents reported a small-to-moderate social network did not differ in their symptoms of depression and anxiety might be related to specific contextual factors associated with families having a parent with BD. Although a larger social network is expected to increase the availability of social support, network size and quality of support are two distinct characteristics that do not necessarily go hand in hand (Cochran and Niego 2002; Gottlieb and Bergen 2010). A person’s social network refers to the structure of their social contacts, whereas their perceived social support refers to their beliefs about the amount and quality of support received from their social contacts (Gottlieb et al., 2010). A larger social network does not necessarily provide adequate social support, especially for individuals with mental disorders who are likely to surround themselves with similar others struggling with mental illness, which may negatively impact their psychological functioning (Schenk et al. 2021). Moreover, individuals with mental illnesses such as BD tend to have poorer interpersonal functioning than those with no mental disorder, and having more social contacts may increase the frequency of their interpersonal conflicts (Eidelman et al. 2012; Walker et al. 1993). Furthermore, parents with BD, relative to parents with no affective disorder, are more likely to select intimate partners that can hinder, rather than help, the family environment and functioning (Serravalle et al. 2020). Intimate partners of adults with BD, relative to partners of adults with no affective disorder, have more mental disorders, higher neuroticism, lower extraversion, more emotion-focused coping, and report higher levels of verbal aggression towards their partners (Serravalle et al. 2020). Therefore, if parents with BD are more likely to be surrounded by extended family, spouses, friends, and acquaintances that can negatively influence their psychological functioning and family environment, having a much larger social network could further aggravate these negative effects. These problematic influences in the parents’ network may then negatively impact the OBD directly through the interactions they have with the individuals in their parents’ social network and indirectly through the effects these relationships have on the parents’ mental health, rearing practices and parent-child bonding (Cochran et al., 2002; Iacono et al. 2018; Lau et al. 2018; Schenk et al. 2021). Conversely, the OBD whose parents reported small-to-moderate social networks may have been exposed to less potentially negative influences and to more optimal family functioning. Research on changes of social networks across the lifespan suggest that as individuals get older, their social networks get smaller and are composed of less peripheral and more close connections, particularly following transitional life events such as becoming a parent (Wrzus et al. 2013). Thus, it is possible that the quality of relationships of the parents in families with a parent having BD with small-to-moderate social networks was greater than of those with the largest social networks.

Contrary to our hypotheses, parents’ use of more task-oriented and less emotion-oriented coping during their children’s middle childhood did not influence the development of depression and anxiety or SUD symptoms in late adolescence and young adulthood among the OBD. The coping style of parents with BD has been found to influence their own mental well-being and the level of family stress (Fletcher et al. 2013), as well as their offspring’s psychosocial functioning during middle childhood (Ellenbogen et al., 2004). However, the present findings indicate that these effects may not play a role in the OBD’s development of psychopathology over time. Given that there is evidence that the OBD adopt more ineffective coping skills as they grow up (Jones et al. 2006; Nijjar et al. 2014), mental health outcomes among the offspring may be influenced by their own coping strategies rather than those of their parents. Unfortunately, the hypothesis that the offspring’s coping strategies mediated the link between parents’ coping and offspring mental health was not assessed in the present study. Moreover, it is possible that focusing on the type of coping that parents engaged in (i.e., task-oriented and emotion-oriented) may not be the most accurate or effective way of assessing the quality of their coping style. In fact, researchers suggest that the quality of a coping strategy varies depending on the type of stressor, and that coping effectiveness might be better understood by assessing coping flexibility. That is, a person’s ability to adjust their coping strategies to meet the demands of different stressors might be more important that fixed coping strategies (Kato 2012). High coping flexibility has been linked to better psychological outcomes (Cheng et al. 2014) and would therefore be worth measuring for future research with similar samples.

The present study is the first longitudinal assessment of the protective effects of parents’ social support and coping practices on the OBD’s mental health outcomes. There are nonetheless study limitations. First, the sample in middle childhood was characterized by a large age range. Social support might have had different effects in older versus younger children. Second, the measures of social support and coping included in this study were limited to parents’ self-report. They are thus limited to the perspective of the parents and do not provide objective information about the quality and frequency of social contacts, which may be important to consider when interpreting these results. However, the present study included assessments of coping and social support by multiple parents in a family, compared to other studies using only a single parent report (e.g., Nunes et al. 2021). Third, the assessment of parents’ coping strategies and social support at a single time point, when their children were in middle childhood, limits our conclusions regarding the timing of the reported parent effects on offspring outcomes. That is, it is not known whether the longitudinal relationship between social support in parents and psychiatric symptoms in young adult offspring was due to effects in middle childhood or continuing social support problems in parents when their offspring were in early adulthood. Fourth, multiple moderation models for each of the two outcomes were conducted, yielding a higher probability of obtaining a false positive result. A priori hypotheses defined the models in this study, but future research that aims to examine similar models may benefit from using a data-driven approach such as a penalized regression analysis, to identify the most robust predictors prior to defining the models. Fifth, recent studies have found poor family functioning in both families having a parent with BD and families having a parent with other mental disorders (Shalev et al. 2019; Stapp et al. 2020). Thus, it is possible that the present findings are not specific to families with a parent having BD. Sixth, although the 24% attrition from time 1 to time 2 is deemed to be statistically acceptable from attrition simulation studies (see Gustavson et al. 2012), it is higher than other longitudinal studies, such as the Dunedin Multidisciplinary Health and Development Study in which attrition was 10% after 11 years (i.e., Poulton et al. 2015), and may have introduced bias in the sample. Lastly, the study sample is mostly middle-class and French Canadian; thus the findings might not generalize to a more diverse population of families with a parent having BD.

Taken together, these findings provide evidence that social support satisfaction in parents, but not social network size or coping strategies, acts as a protective factor against the development of substance use problems in the OBD. This is particularly important, as there is evidence that substance use problems are a substantial negative outcome among the OBD, increasing the risk for future affective disorders (Duffy et al. 2012). Moreover, they show that a larger social network (i.e., number of social contacts), but not social support satisfaction or coping strategies, in families with a parent having BD is associated with an increased risk of depression and anxiety symptoms in their offspring. Future research should assess the quality and type of social support received in parents with BD that have small and large social networks, in order to better understand the mechanisms behind the effects observed in the current study. Overall, these results raise awareness about the environmental factors in parents with BD that may buffer or exacerbate their offspring’s risk of developing adverse mental health outcomes. These findings have implications for the development and improvement of intervention and prevention strategies for the offspring of families having a parent with BD. In addition to current prevention strategies for the OBD which focus on the functioning of the nuclear family (Miklowitz et al. 2020; Resendes et al. 2023; Serravalle et al. 2021), it would be important to promote general and good quality social support from extended family, friends, and the community, since they may provide further protective value against the development of mental health problems for these high-risk children.

### Electronic supplementary material

Below is the link to the electronic supplementary material.

Supplementary Material 1

## Data Availability

The datasets generated and/or analyzed during the current study are not publicly available due to the possibility that individual privacy could be compromised but are available from the corresponding author on reasonable request.
