# Supplementary material for: The perceived social support of parents having bipolar disorder impacts their children’s mental health: a 10-year longitudinal study
Source: Int J Bipolar Disord. 2024 Jul 27;12:27. doi: 10.1186/s40345-024-00349-4 (PMC11283441; doi:10.1186/s40345-024-00349-4)
Supplement: Supplementary file 1 — Supplementary Material 1 [file 40345_2024_349_MOESM1_ESM.docx]

**Supplementary Materials**

**Risk Status**

(OBD vs Control)

**Parent Psychosocial Variables at time 1**

- Social support satisfaction
- Social network size
- Task-oriented coping
- Emotion-oriented coping

**Offspring Mental Health Outcomes at time 2**

- Depression & Anxiety symptoms
- SUD symptoms

**Figure S1.**

Moderation model of the effect of offspring risk status (X) on offspring psychopathology symptoms at time 2 (Y) as moderated by parents’ psychosocial variables at time 1 (W)

**Table S1.**

*Results of ordinary least squares (OLS) regression model predicting offspring depression and anxiety symptoms at time 2 (Y) from offspring risk status (X), parents’ social support satisfaction at time 1 (W) and the X by W interaction term*

| Model | Estimate (*b*) | | *SE* | 95% CI | | *p* |
| --- | --- | --- | --- | --- | --- | --- |
|  |  |  | | *LL* | *UL* |  |
| Risk status (**X**) | .552 | .576 | | -.588 | 1.69 | .34 |
| Parents’ social support satisfaction (**W**) | .043 | .189 | | -.331 | .418 | .82 |
| Offspring age | .154 | .146 | | -.134 | .443 | .291 |
| Mean parent education | .105 | .204 | | -.299 | .507 | .609 |
| Offspring time 1 psychiatric symptoms*^a^* | .251 | .073 | | .106 | .396 | .001* |
| **X** by **W** interaction | -.151 | .19 | | -.528 | .225 | .428 |

*Note*. * *p* < .05.

*LL* = lower limit; *UL* = upper limit

*a* Number of clinical psychiatric symptoms reported by parents on the Child Assessment Schedule

**Table S2.**

*Results of ordinary least squares (OLS) regression model predicting offspring substance use disorder symptoms at time 2 (Y) from offspring risk status (X), parents’ social network size at time 1 (W) and the X by W interaction term*

| Model | Estimate (*b*) | | *SE* | 95% CI | | *p* |
| --- | --- | --- | --- | --- | --- | --- |
|  |  |  | | *LL* | *UL* |  |
| Risk status (**X**) | .574 | .538 | | -.489 | 1.64 | .287 |
| Parents’ social network size (**W**) | -.12 | .06 | | -.238 | .001 | .048* |
| Offspring age | .252 | .143 | | -.031 | .535 | .081 |
| Mean parent education | -.259 | .205 | | -.664 | .146 | .204 |
| Offspring time 1 psychiatric symptoms*^a^* | .038 | .071 | | -.103 | .179 | .591 |
| **X** by **W** interaction | -.089 | .059 | | -.205 | .027 | .132 |

*Note*. * *p* < .05.

*LL* = lower limit; *UL* = upper limit

*a* Number of clinical psychiatric symptoms reported by parents on the Child Assessment Schedule

**Table S3**

*Results of ordinary least squares (OLS) regression model predicting offspring depression and anxiety symptoms at time 2 (Y) from offspring risk status (X), parents’ social network size at time 1 (W) and the X by W interaction term*

| Model | Estimate (*b*) | | *SE* | 95% CI | | *p* |
| --- | --- | --- | --- | --- | --- | --- |
|  |  |  | | *LL* | *UL* |  |
| Risk status (**X**) | .590 | .593 | | -.582 | 1.76 | .321 |
| Parents’ social network size (**W**) | .056 | .062 | | -.067 | .178 | .371 |
| Offspring age | .135 | .146 | | -.154 | .423 | .357 |
| Mean parent education | .044 | .213 | | -.378 | .466 | .837 |
| Offspring time 1 psychiatric symptoms*^a^* | .229 | .073 | | .085 | .373 | .002* |
| Parent’s task-oriented coping score | .061 | .083 | | -.102 | .225 | .458 |
| Parents’ emotion-oriented coping score | .053 | .079 | | -.103 | .210 | .502 |
| Parents’ social support satisfaction | -.050 | .190 | | -.425 | .326 | .794 |
| **X** by **W** interaction | .143 | .060 | | .024 | .262 | .019* |

*Note*. * *p* < .05.

*LL* = lower limit; *UL* = upper limit

*a* Number of clinical psychiatric symptoms reported by parents on the Child Assessment Schedule

**Table S4.**

*Results of ordinary least squares (OLS) regression model predicting offspring substance use disorder symptoms at time 2 (Y) from offspring risk status (X), parents’ social support satisfaction at time 1 (W) and the X by W interaction term*

| Model | Estimate (*b*) | | *SE* | | 95% CI | | *p* |
| --- | --- | --- | --- | --- | --- | --- | --- |
|  |  |  | | *LL* | | *UL* |  |
| Risk status (**X**) | .654 | .580 | | -.494 | | 1.80 | .262 |
| Parents’ social support satisfaction (**W**) | -.134 | .191 | | -.511 | | .244 | .485 |
| Offspring age | .242 | .142 | | -.040 | | .524 | .092 |
| Mean parent education | -.243 | .211 | | -.661 | | .174 | .251 |
| Offspring time 1 psychiatric symptoms*^a^* | .062 | .071 | | -.079 | | .204 | .386 |
| Parent’s task-oriented coping score | .069 | .081 | | -.091 | | .228 | .397 |
| Parents’ emotion-oriented coping score | .011 | .078 | | -.143 | | .164 | .893 |
| Parents’ social network size | -.086 | .056 | | -.197 | | .025 | .127 |
| **X** by **W** interaction | -.443 | .187 | | -.812 | | -.073 | .019* |

*Note*. * *p* < .05.

*LL* = lower limit; *UL* = upper limit

*a* Number of clinical psychiatric symptoms reported by parents on the Child Assessment Schedule

**Table S5.**

*Results of ordinary least squares (OLS) regression model predicting offspring depression and anxiety symptoms at time 2 (Y) from offspring risk status (X), parents’ social support satisfaction at time 1 (W), parents’ mean social and occupational functioning (Z) and the X by W by Z interaction term*

| Model | Estimate (*b*) | | *SE* | | 95% CI | | *p* |
| --- | --- | --- | --- | --- | --- | --- | --- |
|  |  |  | | *LL* | | *UL* |  |
| Risk status (**X**) | .577 | .704 | | -.817 | | 1.97 | .414 |
| Parents’ social support satisfaction (**W**) | -.032 | .244 | | -.515 | | .451 | .896 |
| Parents’ mean SOFAS rating*^a^* | -.106 | .094 | | -.291 | | .079 | .259 |
| Offspring age | .073 | .153 | | -.230 | | .376 | .635 |
| Mean parent education | -.036 | .238 | | -.507 | | .434 | .879 |
| Offspring time 1 psychiatric symptoms*^b^* | .263 | .074 | | .116 | | .411 | .001* |
| **X** by **W** by **Z** interaction | -.057 | .034 | | -.125 | | .010 | .096 |

*Note*. * *p* < .05.

*LL* = lower limit; *UL* = upper limit

*a* SOFAS: social and occupational functioning assessment scale

*b* Number of clinical psychiatric symptoms reported by parents on the Child Assessment Schedule

**Table S6.**

*Results of ordinary least squares (OLS) regression model predicting offspring depression and anxiety symptoms at time 2 (Y) from offspring risk status (X), parents’ social network size at time 1 (W), parents’ mean social and occupational functioning (Z) and the X by W by Z interaction term*

| Model | Estimate (*b*) | | *SE* | | 95% CI | | *p* |
| --- | --- | --- | --- | --- | --- | --- | --- |
|  |  |  | | *LL* | | *UL* |  |
| Risk status (**X**) | .523 | .717 | | -.897 | | 1.94 | .468 |
| Parents’ social network size (**W**) | -.116 | .105 | | -.324 | | .092 | .273 |
| Parents’ mean SOFAS rating*^a^* | .068 | .096 | | -.257 | | .121 | .479 |
| Offspring age | .084 | .151 | | -.215 | | .383 | .578 |
| Mean parent education | .044 | .226 | | -.404 | | .491 | .847 |
| Offspring time 1 psychiatric symptoms*^b^* | .227 | .072 | | .084 | | .371 | .002* |
| **X** by **W** by **Z** interaction | -.025 | .013 | | -.051 | | .001 | .056 |

*Note*. * *p* < .05.

*LL* = lower limit; *UL* = upper limit

*a* SOFAS: social and occupational functioning assessment scale

*b* Number of clinical psychiatric symptoms reported by parents on the Child Assessment Schedule

**Table S7.**

*Results of ordinary least squares (OLS) regression model predicting offspring substance use disorder symptoms at time 2 (Y) from offspring risk status (X), parents’ social support satisfaction at time 1 (W), parents’ mean social and occupational functioning (Z) and the X by W by Z interaction term*

| Model | Estimate (*b*) | | *SE* | | 95% CI | | *p* |
| --- | --- | --- | --- | --- | --- | --- | --- |
|  |  |  | | *LL* | | *UL* |  |
| Risk status (**X**) | .545 | .690 | | -.820 | | 1.91 | .431 |
| Parents’ social support satisfaction (**W**) | -.207 | .239 | | -.680 | | .266 | .389 |
| Parents’ mean SOFAS rating*^a^* | -.107 | .092 | | -.288 | | .075 | .247 |
| Offspring age | .202 | .150 | | -.095 | | .499 | .181 |
| Mean parent education | -.346 | .233 | | -.807 | | .115 | .140 |
| Offspring time 1 psychiatric symptoms*^b^* | .064 | .073 | | -.081 | | .208 | .384 |
| **X** by **W** by **Z** interaction | -.036 | .033 | | -.102 | | .030 | .283 |

*Note*. * *p* < .05.

*LL* = lower limit; *UL* = upper limit

*a* SOFAS: social and occupational functioning assessment scale

*b* Number of clinical psychiatric symptoms reported by parents on the Child Assessment Schedule

**Table S8.**

*Results of ordinary least squares (OLS) regression model predicting offspring substance use disorder symptoms at time 2 (Y) from offspring risk status (X), parents’ social network size at time 1 (W), parents’ mean social and occupational functioning (Z) and the X by W by Z interaction term*

| Model | Estimate (*b*) | | *SE* | | 95% CI | | *p* |
| --- | --- | --- | --- | --- | --- | --- | --- |
|  |  |  | | *LL* | | *UL* |  |
| Risk status (**X**) | .237 | .724 | | -1.20 | | 1.67 | .744 |
| Parents’ social network size (**W**) | -.174 | .106 | | -.384 | | .036 | .104 |
| Parents’ mean SOFAS rating*^a^* | -.082 | .097 | | -.273 | | .110 | .400 |
| Offspring age | .242 | .152 | | -.060 | | .544 | .115 |
| Mean parent education | -.244 | .228 | | -.695 | | .208 | .287 |
| Offspring time 1 psychiatric symptoms*^b^* | .039 | .073 | | -.106 | | .183 | .597 |
| **X** by **W** by **Z** interaction | -.007 | .013 | | -.033 | | .019 | .605 |

*Note*. * *p* < .05.

*LL* = lower limit; *UL* = upper limit

*a* SOFAS: social and occupational functioning assessment scale

*b* Number of clinical psychiatric symptoms reported by parents on the Child Assessment Schedule
